# Combined modalities of resistance in an oxaliplatin-resistant human gastric cancer cell line with enhanced sensitivity to 5-fluorouracil

**DOI:** 10.1038/sj.bjc.6603866

**Published:** 2007-07-03

**Authors:** C-C Chen, L-T Chen, T-C Tsou, W-Y Pan, C-C Kuo, J-F Liu, S-C Yeh, F-Y Tsai, H-P Hsieh, J-Y Chang

**Affiliations:** 1 National Institute of Cancer Research, National Health Research Institutes, Taipei 114, Taiwan, ROC; 2 Division of Hematology Oncology, Department of Medicine, Chang Gung Memorial Hospital-Chiayi, Tao-Yuan 333, ROC; 3 Graduate Institute of Clinical Medical Sciences, Chang Gung University, Tao-Yuan 333, ROC; 4 Department of Internal Medicine, Kaohsiung Medical University Hospital, Kaohsiung 807, ROC; 5 Division of Environmental Health and Occupational Medicine National Health Research Institutes, Zhunan 350, ROC; 6 Division of Biotechnology and Pharmaceutical Research, National Health Research Institutes, Zhunan 350, ROC; 7 Division of Hematology/Oncology, Tri-Service General Hospital, National, Defense Medical Center, Taipei 114, Taiwan, ROC

**Keywords:** oxaliplatin resistance, copper transporter ATP7A, DNA repair, gastric carcinoma, 5-fluorouracil, thymidylate synthase

## Abstract

To identify mechanisms underlying oxaliplatin resistance, a subline of the human gastric adenocarcinoma TSGH cell line, S3, was made resistant to oxaliplatin by continuous selection against increasing drug concentrations. Compared with the parental TSGH cells, the S3 subline showed 58-fold resistance to oxaliplatin; it also displayed 11-, 2-, and 4.7-fold resistance to *cis*-diammine-dichloroplatinum (II) (CDDP), copper sulphate, and arsenic trioxide, respectively. Interestingly, S3 cells were fourfold more susceptible to 5-fluorouracil-induced cytotoxicity due to downregulation of thymidylate synthase. Despite elevated glutathione levels in S3 cells, there was no alteration of resistant phenotype to oxaliplatin or CDDP when cells were co-treated with glutathione-depleting agent, l-buthionine-(*S*,*R*)-sulphoximine. Cellular CDDP and oxaliplatin accumulation was decreased in S3 cells. In addition, amounts of oxaliplatin- and CDDP–DNA adducts in S3 cells were about 15 and 40% of those seen with TSGH cells, respectively. Western blot analysis showed increased the expression level of copper transporter ATP7A in S3 cells compared with TSGH cells. Partial reversal of the resistance of S3 cells to oxaliplatin and CDDP was observed by treating cell with ATP7A-targeted siRNA oligonucleotides or P-type ATPase-inhibitor sodium orthovanadate. Besides, host reactivation assay revealed enhanced repair of oxaliplatin- or CDDP-damaged DNA in S3 cells compared with TSGH cells. Together, our results show that the mechanism responsible for oxaliplatin and CDDP resistance in S3 cells is the combination of increased DNA repair and overexpression of ATP7A. Downregulation of thymidylate synthase in S3 cells renders them more susceptible to 5-fluorouracil-induced cytotoxicity. These findings could pave ways for future efforts to overcome oxaliplatin resistance.

Platinum drugs represent a class of potent chemotherapeutic agents. Alone or in combination with other drugs, first-generation compound *cis*-diammine-dichloroplatinum (II) (cisplatin, CDDP) is active against cancers of the lung, ovary, bladder, head and neck, oesophagus, cervix, and endometrium, and it is curative for the vast majority of patients with testicular cancer (Einhorn, 1997). Over the past 30 years, a number of analogues have been synthesised to enlarge the spectrum of activity, overcome resistance, and/or reduce toxicity. Oxaliplatin is a third-generation compound; in contrast to CDDP, it contains a bulky diaminocyclohexane (DACH) ring. It differs from other platinum compounds in several ways. First, oxaliplatin exhibits activity against colon carcinoma, a prototypical cancer type that shows primary resistance to CDDP and carboplatin (Misset *et al*, 2000). Second, oxaliplatin consistently demonstrates substantial activity against a variety of CDDP-resistant tumours (Cvitkovic, 1998; Raymond *et al*, 1998). Third, oxaliplatin predominantly produces neurotoxicity rather than nephrotoxicity or myelosuppression (Extra *et al*, 1990).

It is commonly accepted that DNA damage is largely responsible for the cytotoxic properties of platinum compounds. Platinum-induced DNA adduct formation inhibits DNA replication and transcription, leading to antiproliferative effects (Sanderson *et al*, 1996). *In vitro* studies document significant differences between oxaliplatin and CDDP regarding the mechanism of action as well as a diversity in biochemical pathways that protect cells against the agents (Rixe *et al*, 1996). The DACH ligand of oxaliplatin might cause steric hindrance to DNA binding (Scheeff *et al*, 1999), and evidence suggests that fewer platinum-DNA adduct lesions are formed for oxaliplatin than for equimolar CDDP (Woynarowski *et al*, 1998; Raymond *et al*, 2002). Even so, oxaliplatin is typically at least as potent as CDDP in inhibiting growth of cancer cells (Rixe *et al*, 1996). These paradoxical findings suggest that DACH–platinum–DNA adducts more effectively inhibit DNA synthesis.

Cellular resistance to platinum, either intrinsic or acquired, is encountered regularly during cancer treatment and severely limits the drugs' therapeutic potential. Numerous reports have gradually clarified the major pathways involved in CDDP resistance. Cells can become resistant to CDDP through reduced drug uptake, enhanced drug detoxification, augmented DNA repair, and increased tolerance for DNA damage (Desoize and Madoulet, 2002). The mechanisms of oxaliplatin resistance are less well established and should never be considered identical to those underlying CDDP resistance because CDDP chemistry is not necessarily applicable for oxaliplatin. For example, the bulky DACH moiety, which is unique to oxaliplatin, might make platinum–DNA lesions less susceptible to recognition by mismatch repair systems (Vaisman *et al*, 1998).

In our current study, we established an oxaliplatin-resistant subline from a human gastric adenocarcinoma cell line to investigate the biochemical and molecular mechanisms through which cells acquire oxaliplatin resistance. Our results show that combined factors contribute to resistance to oxaliplatin and CDDP. In addition, downregulation of the thymidylate synthase (TS) gene in S3 cells renders them more susceptible to 5-fluorouracil (5-FU)-induced cytotoxicity.

## MATERIALS AND METHODS

### Reagents

Oxaliplatin was obtained from Sanofi (New York, NY, USA). *cis*-Diammine-dichloroplatinum, paclitaxel, 5-FU, l-buthionine-(*S*,*R*)-sulphoximine (BSO), and sodium orthovanadate were purchased from Sigma Chemical Co. (St Louis, MO, USA). Arsenic trioxide was purchased from TTY Biopharm (Taipei, Taiwan). Copper sulphate was purchased from Merck (Darmstadt, Germany). Primary antibodies to proteins were purchased from the following companies: ATP7A (BD Transduction Laboratories, Lexington, KY, USA), ATP7B (Novus, Littleton, CO, USA), excision repair cross complementation-1 (ERCC-1) (BD PharMingen, San Diego, CA, USA), TS and glutathione *S*-transferase-*π* (GST-*π*) (Chemicon, Temecula, CA, USA), *α*-tubulin (Sigma), and X-ray cross complementation-1 (XRCC-1) (Neomarkers, Fremont, CA, USA). Horseradish peroxidase-conjugated secondary antibody was purchased from Santa Cruz Biotechnology (Santa Cruz, CA, USA).

### Establishment of oxaliplatin-resistant cell lines

An established human gastric adenocarcinoma cell line, TSGH, was cultured in minimal essential medium supplemented with 5% fetal bovine serum and 100 U ml^−1^ penicillin, 100 *μ*g ml^−1^ streptomycin, and glutamate in a humidified 5% CO_2_ incubator at 37°C. Oxaliplatin-resistant cells were established from TSGH cells by exposure to increasing concentrations of oxaliplatin. Briefly, TSGH cells were initially incubated in completed medium containing 0.5 *μ*g ml^−1^ oxaliplatin that yielded 40% cell survival for a period of 3–4 weeks, and the cells that proliferated were repeatedly subcultured in completed medium containing increasing concentrations of the drug (a 20% increment each time). Cells that grew exponentially in the presence of 2 *μ*g ml^−1^ oxaliplatin were obtained and subcloned by dilution plating in 48-well plates. Five individual clones were isolated and designated as S1–S5 cells. For maintenance, these subclones were cultured under conditions similar to those used for TSGH, except for a higher concentration of fetal bovine serum (10%) and addition of oxaliplatin (2 *μ*g ml^−1^).

### Growth inhibition assay

Exponentially growing cells were cultured in 24-well plates. Resistant cells were maintained in drug-free medium for 3 days before use. Cells were exposed to various concentrations of drugs for 72 h. The methylene blue dye assay was used to evaluate drug effects on cell growth as described previously (Finlay *et al*, 1984). The drug concentration that inhibited cell growth by 50% (IC_50_) was determined after plotting the percentage of growth relative to untreated control *vs* drug concentration.

### Clonogenic assay

Cells in logarithmic growth phase were cultured in six-well plates (250 cells per well) for one generation (about 27 h). Next, cells were treated with various concentrations of drugs for the indicated times. Cells were then washed with pre-warmed phosphate-buffered saline (PBS) twice and maintained in drug-free complete medium for 9–12 days. At the end of the incubation period, cells were fixed and stained with 50% ethanol containing 0.5% methylene blue for 30 min, then washed with water. The number and size of methylene blue-stained colonies were then recorded and compared with drug-free controls. The assays were carried out in triplicate. Data were expressed as means±standard deviations. The LC_50_ was defined as the drug concentration that produced a 50% decrease in cloning efficiency (ie, 50% lethality).

### Determination of cellular reduced glutathione (GSH) content

Total amounts of cellular GSH were determined using the GSH/GSSG (GSSG, oxidised glutathione) ratio assay kit (Calbiochem-Novabiochem, San Diego, CA, USA) following the manufacturer's protocol.

### Western blot analysis

Crude cellular extracts were prepared for Western blot analysis as described previously (Kuo *et al*, 2004). Detection of immunoreactive signals was accomplished with the Western Blot Chemiluminescent Reagent Plus instrument (Perkin Elmer Life Sciences, Boston, MA, USA).

### Northern blot analysis

Total RNA was isolated from TSGH and S3 cells by the Trizol RNA isolation method (Life Technologies Inc., Grand Island, NY, USA). Ribonucleic acid (20 *μ*g per lane) was subjected to 1.2% agarose formaldehyde gel electrophoresis and transferred to a Hybond N^+^ nylon membrane. Membranes were UV cross-linked to immobilise the RNA. Thymidylate synthase or glyceraldehyde-3-phosphate dehydrogenase (GAPDH) probes were labelled with ^32^P using the random primer labelling kit (Stratagene, La Jolla, CA, USA). For prehybridisation, membranes were placed in Quick hybrid solution (GE Healthcare Bio-Sciences Corp., Piscataway, NJ, USA) at 65°C for 1–2 h. Probes were added and hybridised to RNA overnight. Membranes were then washed three times with a solution of 2 × saline sodium citrate (SSC), 0.1% sodium dodecyl sulphate (SDS) at room temperature, and washed three times again with a solution of 1 × SSC, 0.05% SDS at 50°C. Wrapped membranes were exposed to X-OMAT film at −70°C. The expression mRNA level of TS was calculated as the ratio of the radioactivity in these bands relative to that of the GAPDH bands.

### Semiquantitative reverse transcriptase–polymerase chain reaction (RT–PCR)

Ten micrograms of total RNA extracted with Trizol reagent (Life Technologies Inc.) were treated with DNase and converted to cDNA using the SuperScript II RNase H^−^ Reverse Transcriptase System (Invitrogen, Carlsbad, CA, USA). RT–PCR was performed on a Perkin-Elmer GeneAmp PCR System 2400 (Applied Biosystems, Foster City, CA, USA). Polymerase chain reaction primers and TaqMan™ probes to amplify the copper transport protein 1 (CTR1) gene were designed using Primer Express software version 1.0 (Applied Biosystems), as follows: forward 5′-ATTCGGAGAGAGAGGTGCTA-3′, and reverse 5′-GGAGCAGGAATCACGTCTT-3′. The reaction mixture was preheated at 94°C for 5 min, followed by 30 cycles at 94°C for 1 min, 68°C for 30 s, and 72°C for 1.5 min. Final extension was at 72°C for 7 min. In a separate reaction, GAPDH was amplified as the reference gene.

### Platinum accumulation and platinum–DNA adduct assay

Cells (1 × 10^6^) were plated in 100-mm dishes in media without any drugs and were allowed overnight growth. Cells were left untreated or subjected to different concentrations of CDDP or oxaliplatin for 4 h. After treatment, cells were lysed for protein extraction as previously mentioned. For DNA extraction, cell pellets were treated with a genomic DNA purification kit (Promega, Madison, WI, USA) according to the manufacturer's protocol. Concentration of DNA samples was measured at an optometric density of 260 nm (OD_260 nm_) with a Beckman DU 640 spectrophotometer. Platinum determination was carried out with a Perkin Elmer SCIEX ELAN 6100 ICP–Mass spectrometer (Shelton, CT, USA). The instrumental conditions were as follows: RF power 1.5 kW; gem-tip cross-flow nebuliser with Ryton double-path spray chamber; nebuliser gas flow, 0.99 ml min^−1^; dwell time, 200 ms; repetition, 5 times. Data were acquired at an atomic mass unit (a.m.u.) of 193.693. Quantitative analysis of samples was performed by the use of addition calibration as described in the manufacturer's manual. Addition calibration provides an effective way to measure samples through the use of spiked samples. Multi-element Solution 3 (10 mg l^−1^ Au, Hf, Ir, Pd, Pt, Rh, Ru, Sb, Sn, Te) from Spex CertiPrep (Metuchen, NJ, USA) was used as the platinum standard. Student's *t*-test was used to compare the mean of each group with that of the control. A *P* value <0.05 was considered statistically significant.

### Host cell reactivation assay

Chloramphenicol acetyltransferase (CAT) gene (Promega) was used on platinum-damaged reporter DNA, whereas pSV *β*-galactosidase (*β*-gal) control vector served as an internal control for transfection efficiency. Plasmid DNA was treated with either oxaliplatin or CDDP at 37°C followed by ethanol precipitation. The DNA pellet was washed three times with 70% ethanol, air-dried, and finally resuspended in Tris-EDTA (TE) buffer. Cells (1 × 10^6^ per dish) were seeded in 100-mm dishes and allowed to grow overnight. Before use, the plasmid DNA mixture containing 0.5 *μ*g *β*-gal gene and 1.5 *μ*g CAT gene was condensed with buffer Effectene buffer (buffer EC) and Enhancer (Qiagen, Valencia, CA, USA) according to the manufacturer's instructions. The cells were then transiently transfected with the condensed plasmid DNA mixture. After a 24-h transfection period, cellular extracts were prepared and incubated with ^14^C-labeled chloramphenicol and butyryl CoA, essentially as described by Ginot *et al* (1989). After chromatography, the plate was exposed to X-ray film for an autoradiogram. Band-specific intensity was quantitated using an AlphaImager 2000 system. For each transfection, mock-transfected cells were used as background controls, and non-drug-treated plasmids were used as positive controls.

### Histone H_2_AX phosphorylation

Histone H_2_AX phosphorylation was measured by flow cytometry (Leonce *et al*, 2006). Cells (1 × 10^6^) were plated in 100-mm dishes in media without any drugs and were allowed overnight growth. Cells were exposed to the drugs for 24 h, washed, and fixed by 70% ethanol at −20°C for at least 2 h. Samples were washed with PBS and incubated for 5 min in PBS containing 0.1% Triton X-100 at 0°C. Cells were washed and incubated for 1 h at room temperature with antiphosphohistone H_2_AX (Ser139) murine monoclonal antibody (Upstate Biotechnology, Charlottesville, VA, USA). Cells were washed and incubated for 1 h with Rhodamine-conjugated goat antimouse IgG (Santa Cruz Biotechnology). Cells were washed, resuspended with 500 *μ*l PBS, and analysed by flow cytometry.

### Silencing of TS and ATP7A by small interfering RNA transfection

For TS silencing, siRNA duplexes were designed to target sequences on human TS mRNA corresponding to nt 1058-1077 (5′-GGAUAUUGUCAGUCUUUAGG-3′). The selected sequence is unique to TS as indicated to a sequence search. Small interfering RNA duplexes were obtained from Dharmacon (Lafayette, CO, USA). Each RNA contained two additional 2′-deoxythymidine nts on the 3′ end. In addition, a control siRNA duplex, GL2, was obtained from Dharmacon. The procedure of transfection of siRNA into cells was as described previously (Schmitz *et al*, 2004). For ATP7A silencing, human ATP7A siRNA ON-TARGET plus SMART pool (mixed four duplex: CAGAAACGAUAAUACGAUUUU; GAUAAACGCUCCCUAAACAUU; GGUAUUAGCUGUAAAGUCAUU; GCAAAGGAGUCCAUCAUAUUU, Dharmacon) has been used in this study. Negative control was pool of four nontargeting siRNAs: UGGUUUACAUGUCGACUAA; UGGUUUACAUGUUUUCUGA; UGGUUUACAUGUUUUCCUA; UGGUUUACAUGUUGUGUGA, Dharmacon). In brief, TSGH cells in exponential growth phase were plated in six-well plates at 8 × 10^5^ cells per well, grown for 24 h, then transfected with TS siRNA duplex and ATP7A siRNA ON-TARGET plus SMART pool using cationic lipid oligofectamine (Invitrogen), respectively, as described by the manufacturer's instructions. Silencing was examined 0–72 h after transfection with siRNA-oligofectamine complexes. In addition, a control siRNA duplex, GL2, was obtained from Dharmacon. For growth inhibition assay, TSGH cells were plated in six-well plates at 5 × 10^4^ cells per well for overnight. Small interfering RNA-oligofectamine complexes were then added to the wells for the indicated times. Then tested drugs were added to the wells at the indicated concentrations. The methylene blue dye assay was used to evaluate drug effects on cell growth after an additional 72-h (Finlay *et al*, 1984).

## RESULTS

### Establishment of a cellular model for oxaliplatin resistance

To compare sensitive and resistant phenotypes in the context of the same genetic background, we derived sublines from human gastric adenocarcinoma cell line TSGH that are resistant to oxaliplatin. After the TSGH cells have been continuously grown in the presence of step-wise increasing concentrations of oxaliplatin, five resistant clones (S1–S5) were isolated. Those resistant clones possessed similar doubling time when compared to parental cells that is approximately 27 h. The stability of oxaliplatin resistance in those five sublines was determined by assessing sensitivity after a period of oxaliplatin-free culturing. Growth inhibition assays conducted 3, 14, and 21 days after oxaliplatin withdrawal showed no changes in drug sensitivity. Because the sensitivity to oxaliplatin was similar among those five clones that are about 55-fold more resistant than the parental cells, thus, we chose the S3 subclone for further studies.

### Assessment of cellular sensitivity of S3 cells to drugs

To evaluate the specificity of the resistance to oxaliplatin, we tested the sensitivity of the S3 cells to several drugs. The IC_50_ values are shown in Table 1. Under our experimental conditions, the S3 cell line was approximately 58- and 11-fold more resistant to oxaliplatin and CDDP, respectively. S3 cells were also cross-resistant to two metalloid-containing agents, copper sulphate and arsenic trioxide. The IC_50_ values of S3 cells were approximately 2- and 4.7-fold higher for copper sulphate and arsenic trioxide, respectively, than those of TSGH cells. Besides, we also found that S3 cells were four times more susceptible to 5-FU. Both cell lines exhibited a similar degree of sensitivity to paclitaxel.

### Role of GSH and GST-*π* in modulation of platinum resistance in S3 cells

**Figure 1:**
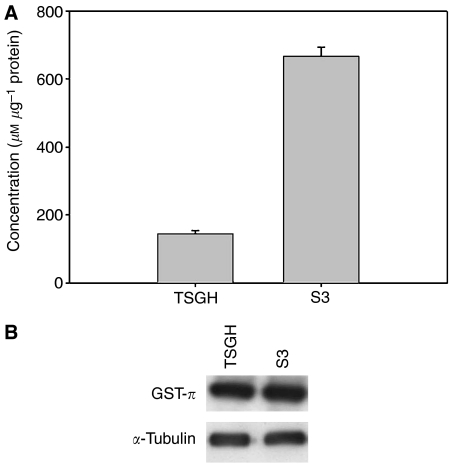
Cellular GSH content and the expression level of GST-*π* in TSGH and S3 cells. (**A**) Total amount of GSH in TSGH and S3 cells was determined using GSH/GSSG ratio assay kit as described in Materials and Methods. The data are the means±s.d. of three independent experiments. (**B**) Analysis of the expression level of GST-*π* by using the Western blot analysis. *α*-Tubulin has been used as internal control. The results are the representatives of at least three independent experiments.

Reduced glutathione has been addressed as an important determinant for platinum resistance. In addition, GST-*π* has been addressed as a xenobiotic metabolising enzyme involved in the detoxification of platinum derivatives. Therefore, the cellular GSH content and the expression level of GST-*π* in both parental and S3 cells were determined. As shown in Figure 1A, S3 cells displayed about a 4.6-fold higher cellular level of GSH. However, there was no difference in the expression level of GST-*π* between resistant and parental cells (Figure 1B).

To clarify further whether the elevated GSH level played a role in development of platinum resistance in S3 cells, we treated S3 cells with either oxaliplatin or CDDP in the presence or absence of GSH-depleting agent BSO for the indicated times and evaluated the result by clonogenic assay. As shown in Table 2, no alteration of resistance to either oxaliplatin or CDDP was noted after GSH depletion.

### Changes in the cellular accumulation of platinum, formation of platinum–DNA adducts, and histone H_2_AX phosphorylation in S3 cells

**Figure 2:**
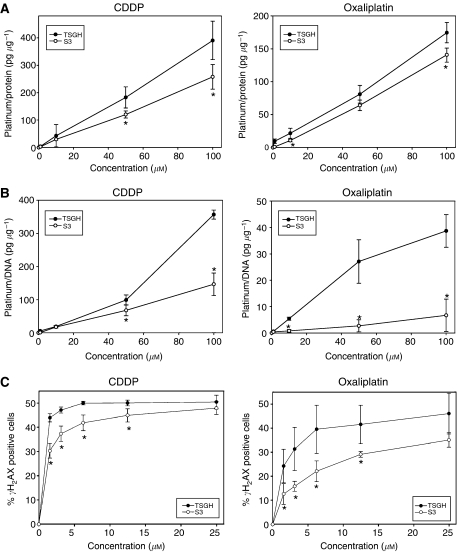
Cellular platinum accumulation, platinum-DNA adducts formation, and histone H_2_AX phosphorylation between TSGH and S3 cells. (**A**) Cellular platinum accumulation and (**B**) Platinum–DNA adducts formation of TSGH and S3 cell lines after exposure to various concentrations (1, 10, 50, 100 *μ*M) of either CDDP or oxaliplatin. Each data points are means from three independent experiments. (**C**) Induction of histone H_2_AX phosphorylation. TSGH and S3 cells were exposed for 24 h to the indicated concentrations of platinum drugs, fixed and labelled with an antiphosphohistone H_2_AX monoclonal antibody before flow cytometric analysis. *γ*H_2_AX positive cells were quantified and expressed as a function of drug concentration. Error bars show the standard deviations. ^*^ (*P*<0.05), significantly different for platinum accumulation, DNA adduct formation, and histone H_2_AX phosphorylation between TSGH and S3 cells at equimolar concentration of platinum drugs.

To determine whether the differences in sensitivity to platinum drugs between TSGH and S3 cells were accompanied by discrepancies in whole platinum accumulation, both cells were exposed to various concentrations of oxaliplatin or CDDP for 4 h. As shown in Figure 2A, our data demonstrates that cellular accumulation of both oxaliplatin and CDDP increases in a dose-dependent manner. At equimolar concentrations, there was a significantly higher accumulation of platinum after CDDP exposure than after oxaliplatin exposure in both TSGH and S3 cells. Besides, there were also significant differences in accumulation of oxaliplatin and CDDP between resistant and sensitive cell lines. The accumulation of CDDP in S3 cells at the concentration of 100 *μ*M was about 35% less than that seen in TSGH cells. S3 cells also showed less accumulation of oxaliplatin than that of TSGH cells, but the decrement level was only about 12% at the concentration of 100 *μ*M (Figure 2A).

Because of changes in cellular accumulation of platinum between TSGH and S3 cells after oxaliplatin or CDDP treatment, we therefore examined the level of DNA platination in those paired cells. The total platinum/DNA adduct levels after a 4-h exposure to oxaliplatin or CDDP are shown in Figure 2B. Comparison of the platinum/DNA level between oxaliplatin and CDDP indicated that at equimolar concentration, there was less platinum bound to DNA after oxaliplatin exposure than after CDDP exposure in both TSGH and S3 cells. There were also significant differences in the amount of DNA-bound platinum between resistant and sensitive cell lines. As seen in Figure 2B, the amounts of oxaliplatin–DNA and CDDP–DNA adducts in S3 cells were about 85 and 60% less than those in TSGH cells at the concentration of 100 *μ*M oxaliplatin and CDDP, respectively.

Because more platinum–DNA adducts were formed in TSGH cells than in S3 cells after platinum treatment, we would like to determine the frequency of DNA double-strand breaks (DSBs) in both cells. We found that the levels of *γ*H_2_AX, a marker of DSBs, were identical between TSGH and S3 cells in untreated condition (data not shown). After oxaliplatin and CDDP treatment, the level of *γ*H_2_AX in TSGH cells was notably higher than that in S3 cells (Figure 2C).

### ATP7A plays a role in modulation of platinum resistance in S3 cells

**Figure 3:**
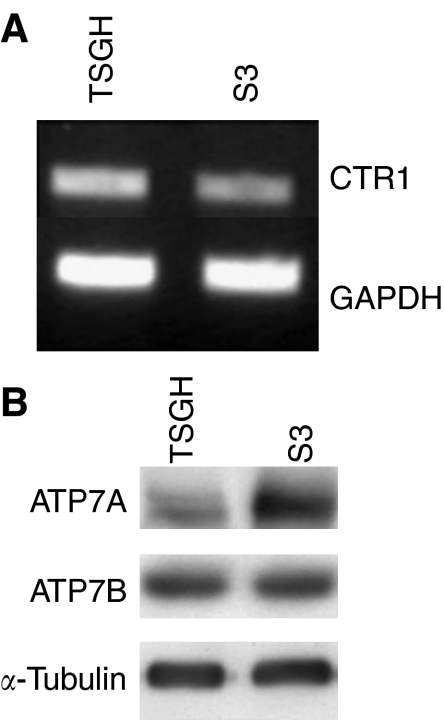
Determination of the expression level of the copper transporters between TSGH and S3 cell. (**A**) Analysis of the expression level of copper uptake transporter CTR1 by using semiquantitative RT–PCR. GAPDH has been used as internal control. The results are the representatives of at least three independent experiments. (**B**) Analysis of the expression level of copper efflux transporters ATP7A and ATP7B by using Western blot analysis. *α*-Tubulin has been used as internal control. The results are the representatives of at least three independent experiments.

Copper homeostasis plays an important role in the uptake and efflux of the platinum drugs. Because of changes in the cellular accumulation of oxaliplatin and CDDP in S3 cells, we therefore evaluated the expression level of copper transporters to check whether they were involved in platinum resistance or not. CTR1 is a main copper uptake transporter in human cells. Our result showed that the expression level of CTR1 mRNA in S3 cells was about 20% less than that in parental cells by quantitating band-specific intensity after adjusting the intensity of GAPDH, the internal standard (Figure 3A). There was no difference in expression levels of copper efflux transporter ATP7B between parental and resistant cell lines in protein level (Figure 3B). However, the expression of copper efflux transporter ATP7A was about threefold higher in S3 cells than in parental TSGH cells (Figure 3B).

**Figure 4:**
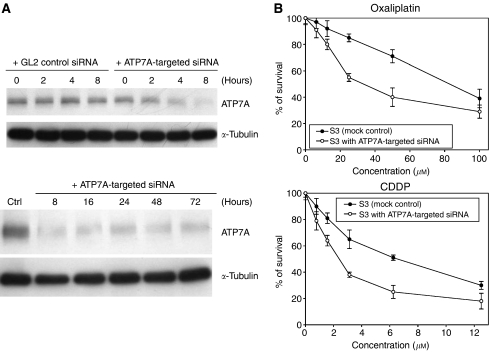
Effect of ATP7A-targeted siRNA on sensitivity of S3 cells towards oxaliplatin and CDDP. (**A**) Western blot analysis of S3 cells after treatment with an ATP7A-targeted siRNA. Cells were incubated in the absence or presence of ATP7A siRNA ON-TARGET plus SMART pool for the indicated times, and harvested and lysed for Western blot analysis. *α*-Tubulin as internal control. (**B**) Effect of ATP7A-targeted siRNA on sensitivity of S3 cells towards oxaliplatin and CDDP. S3 cells were transiently transfected with ATP7A-targeted siRNA for 9 h, then cells were exposed to oxaliplatin and CDDP for another 72 h. Cell growth was determined by methylene blue dye assay. Each value represents the mean of three independent experiments.

To further confirm the role of ATP7A in platinum resistance, a P-type ATPase inhibitor, sodium orthovanadate, was used to determine whether inhibition of ATP7A function could reverse the phenotype of drug resistance. TSGH and S3 cells underwent a 1-h pretreatment with orthovanadate followed by a 72-h treatment with different concentrations of oxaliplatin and CDDP. As shown in Table 3, orthovanadate was able to partially reverse oxaliplatin and CDDP resistance in S3 cells. In addition, we also used RNA interference system to confirm the role of ATP7A in platinum resistance. As shown in Figure 4A, treatment of S3 cells with ATP7A-targeted siRNA was quite effective, resulting in significant knockdown of cellular ATP7A protein level. The expression of control protein, *α*-tubulin, was unaffected by ATP7A-targeted siRNA treatment (Figure 4A). Treatment with a control GL2 siRNA had absolutely no effect on levels of ATP7A or *α*-tubulin.

Consistently, S3 cells transfected with ATP7A-targeted siRNA were approximately threefold more sensitive to oxaliplatin and CDDP (Figure 4B). These results indicated that ATP7A plays a role in modulation of platinum resistance in S3 cells.

### Increased DNA-repair capacity in S3 cells

**Figure 5:**
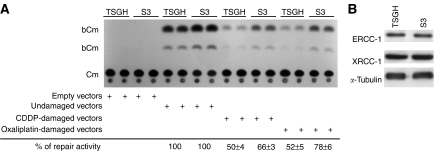
DNA repair ability between TSGH and S3 cells. (**A**) Enhanced DNA repair capacity of CDDP- and oxaliplatin-induced DNA damage by host cell reactivation assay. Percent of repair activity was measured using the undamaged vectors as control. This result is the representative of three independent experiments (mean±s.d.). (**B**) Evaluation of the DNA repairing proteins. The expression levels of ERCC-1 and XRCC-1 were examined in TSGH and S3 cells by Western blot analysis. *α*-Tubulin has been used as internal control. The results are the representatives of at least three independent experiments.

After platinum has formed a large number of adducts on DNA and converted to DSBs, cell death is still not certain, because cells with increased DNA repair may be fixing those lesions. To address the possibility that platinum/DNA adducts are less prone to removal by repair processes, we analysed the reactivation of oxaliplatin- or CDDP-treated plasmids in both TSGH and S3 cells. As shown in Figure 5A, enhanced DNA-repair capacity was found in S3 cells transfected with either oxaliplatin- or CDDP-damaged plasmids. After correction with transfection efficiency determined by *β*-gal enzyme activity, the DNA repair capacity for oxaliplatin and CDDP damage in S3 cells was about 26 and 16%, respectively, higher than in TSGH cells.

Deoxyribonucleic acid repair after platinum adducts' formation involves nucleotide excision repair (NER), for which ERCC-1 is a critical component. In addition, XRCC1 may also be involved in the repair of other types of DNA damage caused by platinum such as double-stranded breaks. However, our result demonstrated that there were no differences in expression levels of ERCC-1 and XRCC-1 between TSGH and S3 cells (Figure 5B).

### Enhanced sensitivity to 5-FU correlated with TS downregulation in S3 cells

**Figure 6:**
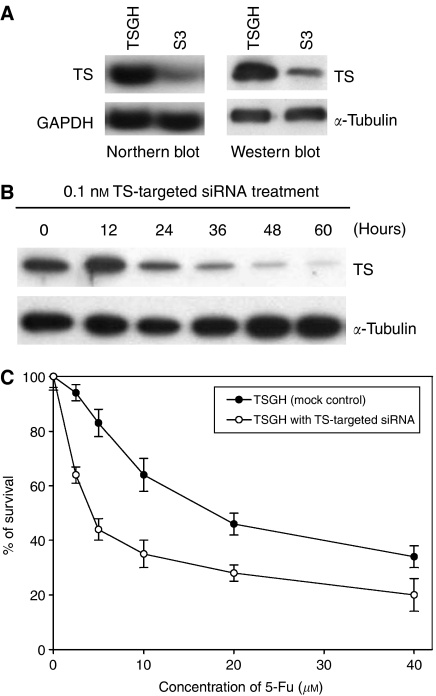
Enhanced sensitivity to 5-FU correlated with TS downregulation in S3 cells. (**A**) Analysis of TS protein and mRNA levels in TSGH and S3 cells by Western blotting and Northern blotting, respectively. *α*-Tubulin has been used as internal control in Western blotting. Beside, GAPDH has been used as internal control in Northern blotting. The results are the representatives of at least three independent experiments. (**B**) Western blot analysis of TSGH cells after treatment with a TS-targeted siRNA. Cells were incubated in the absence or presence of 0.1 nM of TS-targeted siRNA for the indicated times, and harvested and lysed for western blot analysis. (**C**) Effect of TS-targeted siRNA on sensitivity of TSGH cells towards 5-FU. TSGH cells were transiently transfected with TS-targeted siRNA at concentration of 0.1 nM for 12 h, then cells were exposed to 5-FU for another 72 h. Cell growth was determined by methylene blue dye assay. Each value represents the mean of three independent experiments.

It is interesting to know from Table 1 that S3 cells were more susceptible to 5-FU than TSGH cells. We thus examined the expression level of TS protein as well as mRNA in the paired cells. As shown in Figure 6A, the protein level of TS was about four times lower in S3 cells than that in TSGH cells. Northern blot analysis also demonstrated consistent findings.

To further clarify the enhancement of sensitivity to 5-FU in S3 cell whether correlated with downregulation of TS gene, reducing the TS levels in parent TSGH cells via TS-targeted siRNA performed in this study. As seen in Figure 6B, knockdown of TS gene with TS-target siRNA was observed in a time-dependent manner in TSGH cells. The expression of control protein, *α*-tubulin, was unaffected by TS-target siRNA treatment. Treatment with a control GL2 siRNA had absolutely no effect on levels of TS or *α*-tubulin (data not shown). Furthermore, those TS-silencing TSGH cells were 4.2-fold more sensitive to 5-FU than the mock control (IC_50_ 5-FU, 3.9±1.5 *vs* 16.2±3.1 *μ*M) (Figure 6C).

## DISCUSSION

Although evidence of cross-resistance between CDDP and oxaliplatin exists (Rixe *et al*, 1996; Safaei and Howell, 2005), oxaliplatin has been frequently shown to be effective against tumours with primary or acquired resistance to CDDP (Cvitkovic, 1998; Misset *et al*, 2000). Conversely, CDDP is rarely tested in oxaliplatin-resistant cancer cells either *in vivo* or *in vitro*. Hector *et al* (2001) reported that an oxaliplatin-resistant subline from the A2780 ovarian cancer cell line demonstrated impaired accumulation of both CDDP and oxaliplatin, hence cross-resistance to CDDP. Nevertheless, their CDDP-resistant subline exhibited decreased cellular content of CDDP only, but not oxaliplatin. Those differences in cellular handling of CDDP and oxaliplatin raise the question whether mechanisms of resistance between these two drugs are indeed similar. In contrast to extensive studies of the cellular pharmacology of CDDP, little is known about how cells become resistant to oxaliplatin. Recent work has identified pivotal roles of copper transporters in trafficking platinum compounds through cells and controlling their activities in cells (Safaei and Howell, 2005). However, most of our knowledge comes either from CDDP-resistant cell lines or from cell lines manipulated by transfection with these transporters. To improve the knowledge of the molecular basis of resistance to oxaliplatin, we generated an *in vitro* cellular model of resistance derived from the TSGH gastric adenocarcinoma cell line, S3, by exposure to increasing concentrations of oxaliplatin up to 2 *μ*g ml^−1^. According to the data from pharmacokinetic study, the peak level in patients receiving customary 2-h infusion of oxaliplatin at the dose of 85 mg m^−2^ is 0.814 *μ*g ml^−1^ (Graham *et al*, 2000; Simpson *et al*, 2003), which is much lower than the concentration we used (2 *μ*g ml^−1^). However, in patients receiving higher dose (130 mg m^−2^) of oxaliplatin infusion, the *C*_max_ could reach 2.59–3.22 *μ*g ml^−1^ (Graham *et al*, 2000), suggesting that although S3 cell line is highly oxaliplatin-resistant, it is still clinically achievable. In the present study, we performed a parallel comparison of oxaliplatin-resistant S3 cells and their parental counterparts in terms of GSH level, drug accumulation, platinum/DNA adduct formation, and DNA repair for platinum drugs.

Increased intracellular GSH has been previously associated with platinum resistance in many studies (Andrews *et al*, 1985; el-akawi *et al*, 1996). In addition, GST-*π* is a metabolic enzyme which participates in the detoxification of platinum derivatives and is an important mediator of both intrinsic and acquired resistance to platinum (Cullen *et al*, 2003). We determined GSH content and the expression level of GST-*π* in resistant and parental cell lines. The results showed that the level of GSH in S3 cells was 4.6-fold higher than in parental TSGH cells (Figure 1A), but there was no apparent discrepancy in the expression level of GST-*π* protein (Figure 1B). After treating S3 cells with GSH-depleting agent BSO in the presence of platinum drugs, no alteration in drug susceptibility was found (Table 2). This suggests that depletion of GSH is not enough to overcome the oxaliplatin as well as CDDP resistance in this model. The modulation of platinum resistance in S3 cells may be through pathways irrelevant with GSH content.

The results obtained from platinum accumulation assay disclosed that cellular concentration of CDDP and oxaliplatin decreased by about 35 and 12%, respectively, in S3 cells when compared to parental TSGH cells (Figure 2A). The reduced intracellular concentration of platinum drugs can be either due to decreased uptake, increased export, or both. Emerging evidence has shown that mechanisms involved in copper homeostasis play important roles in platinum transportation through cells (Komatsu *et al*, 2000; Ishida *et al*, 2002; Samimi *et al*, 2003). One of the earliest hints that copper homeostasis mechanisms might be involved in resistance to the platinum drugs was the observation that cells resistant to platinum drugs are cross-resistant with other metal or metalloid-containing agents (Naredi *et al*, 1995; Shen *et al*, 1998). In our oxaliplatin model, we found that S3 cells were cross-resistant to copper sulphate and arsenic trioxide (resistance index, 2 and 4.7, respectively) (Table 1). Coupled with findings that cells selected for resistance to platinum drugs are cross-resistant to copper and *vice versa*, copper transporters have gradually gained notice in the search for pathways involved in platinum resistance (Safaei *et al*, 2004).

The major copper influx transporter CTR1 has been shown to assist uptake of CDDP, carboplatin, and oxaliplatin and to regulate their cytotoxicity in yeast and mammalian cells (Ishida *et al*, 2002; Lin *et al*, 2002). However, little is known about whether CTR1 also mediates oxaliplatin pharmacology in human cancer cells or not. Song *et al* (2004) recently showed that transfection of CTR1 into a CDDP-resistant, CTR1-deficient subline of a small-cell lung cancer cell line enhanced cellular uptake of all platinum compounds and increased sensitivity to CDDP and carboplatin, but not to oxaliplatin. Holzer *et al* (2006) demonstrated that CTR1 controls the cellular accumulation of CDDP, carboplatin, and oxaliplatin at low concentrations. However, accumulation of oxaliplatin is not dependent on CTR1 at higher concentrations. In our study, semiquantitative RT–PCR analysis revealed only 20% reduction of CTR1 mRNA level in S3 cells compared with TSGH cells (Figure 3A). Such minor reduction of CTR1 expression implies that CTR1 may not be a major determinant of platinum resistance in S3 cells.

Export of copper from mammalian cells involves two Cu efflux transporters, ATP7A and ATP7B, which belong to P-type ATPase. It has been documented that ATP7A and ATP7B sequestered platinum drugs from the cytoplasm into subcellular compartments, mainly vesicles localized to the trans-Golgi network (TGN) for subsequent efflux in a manner similar to their effects on copper (Klomp *et al*, 1997). This action led to reduced platinum cytotoxicity (Samimi *et al*, 2004). Available information consistently provided strong evidence that ATP7B mediates resistance to platinum drugs by regulating drug efflux, and tumours with higher ATP7B expression did show an unfavourable response to platinum drug treatment (Safaei and Howell, 2005). However, most of these reports explored the association between ATP7B overexpression and CDDP or carboplatin resistance. The only research group that manoeuvred an ATP7B-overexpressing fibroblast cell line actually observed an increased sensitivity to oxaliplatin (Samimi *et al*, 2004). With the expression level of ATP7B in S3 cells similar to that in TSGH cells, our results suggest that ATP7B plays no role in development of oxaliplatin resistance in S3 cells.

Among the three copper transporters, substantially less information is available regarding the ability of ATP7A to modulate cellular pharmacokinetics of the platinum drugs. Study of the cellular pharmacology of copper and CDDP in the ATP7A-deficient cells demonstrated that lack of ATP7A function was associated with increased accumulation of both copper and CDDP, and hypersensitivity to both agents (Samimi *et al*, 2003). When transfected with an ATP7A expression vector, these cells were rendered CDDP resistant (Samimi *et al*, 2003). Overexpression of ATP7A was seen in our oxaliplatin-resistant S3 cells (Figure 3B). To confirm further the role of ATP7A in oxaliplatin resistance in S3 cells, a P-type ATPase inhibitor, sodium orthovanadate, and ATP7A-targeted siRNA have been used to determine whether inhibition of ATP7A function could reverse the phenotype of drug resistance. We found that sodium orthovanadate was able to partially reverse oxaliplatin and CDDP resistance in S3 cells (Table 3). Likewise, S3 cells transfected with ATP7A-targeted siRNA were approximately threefold more sensitive to oxaliplatin and CDDP (Figure 4B). Our data suggest that overexpression of ATP7A in S3 cells results in increased binding and sequestration of platinum drugs, which keeps them away from accessing their key cytotoxic targets in the nucleus. These results indicate that ATP7A plays a role in modulation of platinum resistance in S3 cells.

The role of ATP7A in development of platinum resistance in S3 cells, however, could not be completely validated in our growth-inhibition assays with pretreatment with sodium orthovanadate and ATP7A-targeted siRNA (Table 3 and Figure 4B). The modest reduction in resistance ratios raises the possibility that factors other than ATP7A are involved in mediating oxaliplatin and CDDP resistance in S3 cells. According to our result, the amounts of oxaliplatin–DNA and CDDP–DNA adducts in S3 cells were about 15 and 40% of those seen with TSGH cells, respectively (Figure 2B). Damia *et al* (1998) showed that the ability of cells to repair platinum-induced DNA lesions was an important factor in determining CDDP cytotoxicity. Reardon *et al* (1999) also revealed that both CDDP and oxaliplatin adducts were removed to a similar extent by the excision repair system. Our host cell reactivation assay clearly demonstrated that DNA damage repair was pivotal in increased cellular resistance to oxaliplatin. After transfection, both CDDP- and oxaliplatin-damaged plasmids were repaired more efficiently in S3 cells than in TSGH cells. The increments of DNA repair in S3 cells were about 26 and 16% for oxaliplatin- and CDDP-induced DNA damage, respectively (Figure 5A).

Histone H_2_AX has been implicated in the maintenance of genomic stability by participating in the repair of DNA damage (Davalos and Campisi, 2003; Sengupta *et al*, 2004). The phosphorylation of histone H_2_AX on Ser139 by the ataxia telangiectasia mutated (ATM) and ATR (ATM and Rad 3 related) kinases is an early event observed after the generation of DSBs by ionising radiation or DNA-cross-linking agents (Rogakou *et al*, 1998; Banath and Olive, 2003). Thus, we used phosphorylated histone H_2_AX (*γ*H_2_AX) as a marker to compare the induction of DSBs by oxaliplatin and CDDP to see whether differences existed between TSGH and S3 cells. Consistently, less DSBs was observed in platinum-treated S3 cell (Figure 2C).

Nucleotide excision repair, a network of many proteins gathered in a DNA repair system, is one of the major pathways responsible for drug resistance. Excision repair cross complementation-1 plays a pivotal role in NER pathway, influencing the repair of platinum/DNA damage because of its adducts recognition and excision ability (Lee *et al*, 1993). It has been indicated that subjects with lower ERCC-1 levels had lower DNA repair capacity (Rosell *et al*, 2003). On the other hand, XRCC-1 has been shown to be involved in the repair of other types of DNA damage caused by CDDP including double-stranded breaks (Weaver *et al*, 2005). To gain insights into the molecular basis for enhanced repair in our model system, we measured the expression levels of both ERCC-1 and XRCC-1 in TSGH and S3 cells. Both proteins were not differentially expressed in the paired cell lines (Figure 5B). Further studies might be necessary to determine how the repair system works to protect cancer cells from platinum-induced cytotoxicity by mending DNA lesions.

Other than the mechanism of platinum resistance, it is interesting to find that cells resistant to oxaliplatin and CDDP show enhanced sensitivity to 5-FU in S3 cells (Table 1). Thymidylate synthase is the target enzyme of 5-FU (Peters *et al*, 1994), and it is now well known that decreased TS gene and protein expression correlates with better clinical responsiveness of colorectal and gastric cancers to 5-FU treatment (Johnston *et al*, 1995; Lenz *et al*, 1996). Our TSGH gastric cancer cells that had been rendered oxaliplatin resistant exhibited significantly decreased TS protein level, which resulted in enhanced susceptibility to 5-FU cytotoxicity (Table 1 and Figure 6A, right). The enhanced sensitivity to 5-FU and decreased expression level of TS protein were seen not only in S3 cells, but also in the other selected oxaliplatin-resistant clones, suggesting that change in 5-FU sensitivity/TS status was reproducible in our system (data not shown).

We further demonstrated that downregulation of TS protein expression was the result of decreased level of the corresponding mRNA (Figure 6A, left). Moreover, we also showed that TSGH cells could become more sensitive to 5-FU through gene silencing via TS-targeted siRNA method (Figure 6B), which validated the important role of downregulated TS gene in rendering S3 cells more sensitive to 5-FU (Figure 6C). Although the detailed mechanism for oxaliplatin-induced TS gene downregulation needs to be further elucidated, our result is concordant with two previous reports (Raymond *et al*, 2002; Yeh *et al*, 2004), which indicated that oxaliplatin could downregulate TS in cancer cells and thus potentiate the efficacy of 5-FU. As oxaliplatin and 5-FU have been shown to be highly synergistic not only in preclinical models (Raymond *et al*, 1997) but also in subsequent clinical trials (Rothenberg *et al*, 2003; Chao *et al*, 2004; Lordick *et al*, 2005; Schippinger *et al*, 2005; Cavanna *et al*, 2006), our data provide important information regarding why the combination of oxaliplatin and 5-FU results in better objective response than single use alone.

In conclusion, our study indicates that the mechanisms responsible for oxaliplatin and CDDP resistance in S3 cells are overexpression of copper efflux transporter ATP7A and enhancement of DNA repair capacity. Cells rendered oxaliplatin resistant showed decreased expression of TS gene, which resulted in their greater vulnerability to 5-FU. Collectively, these findings could pave ways for future efforts to overcome oxaliplatin resistance.

## Figures and Tables

**Table 1:** Cell sensitivity to chemotherapeutic drugs and metalloid-containing agents in TSGH and S3 cells

	**IC_50_ values^a^**		
**Drugs**	**TSGH**	**S3**	**RI^b^**
Oxaliplatin (*μ*M)	1.43±0.08^b^	84.1±4.2	58.7
CDDP (*μ*M)	0.58±0.03	6.7±0.2	11.6
Copper sulphate (*μ*M)	167.0±4.3	328.2±19.9	1.97
Arsenic trioxide (*μ*M)	5.0±0.5	23.5±2.9	4.7
5-FU (*μ*M)	15.9±0.5	4.0±0.1	0.25
Paclitaxel (nM)	3.0±1.8	4.2±2.3	1.4

a Cells were treated with various concentrations of test drugs for 3 days. Cell growth was determined by methylene blue dye assay. The IC_50_ value resulting from 50% inhibition of cell growth was calculated. Each value represents the means±s.d. of three independent experiments.

b Resistance index (RI) is calculated as RI=(IC_50_ S3 cells)/(IC_50_ TSGH cells).

**Table 2:** The effect of BSO, a GSH-depleting agent, on cellular susceptibility of TSGH and S3 cells to cisplatin and oxaliplatin cytotoxicity

	**BSO concentration**		
**Cell lines and drugs**	**0**	**50 *μ*M**	**100 *μ*M**
*TSGH*			
CDDP (*μ*M)	0.54±0.05	0.62±0.07	0.61±0.13
Oxaliplatin (*μ*M)	1.51±0.17	1.69±0.21	1.39±0.09
			
*S3*			
CDDP (*μ*M)	6.52±0.3	6.14±0.14	7.01±0.28
Oxaliplatin (*μ*M)	80.2±6.9	70.3±7.5	84.9±8.8

TSGH and S3 cells were treated with platinum drugs in the presence or absence of BSO for 24 h, then cultured in drug-free medium continuously for 10 days. The LC_50_ was defined as the concentration of drug that inhibited colony formation by 50% relative to drug-free controls. Values are means±s.d. of three independent experiments.

**Table 3:** The effect of sodium orthovanadate, a P-type ATPase inhibitor, on cellular susceptibility of TSGH and S3 Cells to cisplatin and oxaliplatin cytotoxicity

	**IC_50_ values^a^**	
**Cell lines and drugs**	**Without sodium orthovanadate treatment**	**10 *μ*M sodium orthovanadate**
*Oxaliplatin*		
TSGH (*μ*M)	1.47±0.15	2.29±0.22
S3 (*μ*M)	85.4±7.2	69.1±2.33
Resistant index^b^	58.1	30.2
		
*CDDP*		
TSGH (*μ*M)	0.56±0.05	0.84±0.10
S3 (*μ*M)	6.8±0.2	5.8±0.3
Resistant index	12.1	6.9

a Both TSGH and S3 cells were pre-treated with 10 *μ*M of sodium orthovanadate for 1 h followed by concurrent exposure to either CDDP or oxaliplatin for 72 h. Cell growth was determined by methylene blue dye assay. The IC_50_ value resulting from 50% inhibition of cell growth was calculated. Each value represents the means±s.d. of three independent experiments.

b Resistance index (RI) is calculated as RI=(IC_50_ S3 cells)/(IC_50_ TSGH cells).
